# Supplementary material for: Congruent evolutionary responses of European steppe biota to late Quaternary climate change
Source: Nat Commun. 2022 Apr 8;13:1921. doi: 10.1038/s41467-022-29267-8 (PMC8993823; doi:10.1038/s41467-022-29267-8)
Supplement: Supplementary file 1 — Supplementary Information [file 41467_2022_29267_MOESM1_ESM.pdf]

Supplementary Information

Congruent evolutionary responses of European steppe biota to late Quaternary climate change

Kirschner, Perez et al.

Correspondence: Philipp Kirschner (*philipp.kirschner@gmail.com*)

Supplementary Methods

CNN based demographic modeling

We evaluated three potential scenarios for the evolution of European steppe biota during the Pleistocene climatic oscillations (Figure 1): 1) Parallel expansion, consisting in the expansion of population sizes for both zonal and extrazonal lineages; 2) Zonal expansion only, with a population size expansion for the Zonal lineage and stable population size in the extrazonal lineage; 3) No expansion, in which population sizes are stable for both lineages. Such scenarios were conceived with basis on SDM projections and stairway plots (Figure 2, Supplementary Figure 2). For each scenario, we performed 10,000 coalescent simulations with the software ms[1], adopting specific priors for each species concerning generation time and mutation rate (as described in the Methods section), as well as for population sizes (based on stairway plots; Supplementary Figure 2). We also used priors for parameters shared by all species, namely the splitting time (T1) between the two lineages in all scenarios, and the time for the first (T2) and second (T3) demographic events in the Parallel expansion and Zonal expansion only scenarios, sampled from an uniform distribution spanning from 110,000 to 2,400,000; 12,000 to 110,000; and 0 to 12,000 years, respectively. We also simulated migration events in both directions and during two different time periods, pre-LGP (between T2 and T1)and the LGP (between T3 and T2) for all models, sampled from uniform distributions from 0 to 5 migrants per generation. Each simulation was carried using a modified version of the scripts from Oliveira et al.[2], with sample sizes according to the empirical dataset of each species and 1,000 SNPs (fixed by using the -s option). We loaded each simulated data as images (NumPy arrays) including samples as columns and loci as lines. In order to train our CNN to recover information from the genotype matrices, while also recognizing missing data, we transformed the simulations by converting the genotypes coded as 0 (reference state in the program ms) to -1, while maintaining the genotypes coded as 1 (alternate state) unchanged. Then, we randomly added missing genotypes (coded as 0s) according to the percentage observed in each species (Table 1). The order of the arrays (simulations) were then shuffled and we separated 25% random simulations to be used as the

validation set, while the remaining 75% were used as the training set. We used the network architecture from Oliveira et al.[2], modified to include the suggestions from Sanchez et al.[3]. The included suggestions were the use of different kernel sizes in the first layers (we applied four 1D kernels of sizes 3, 5, 20 and 50 in the second layer), and the intercalation of convolutional layers with batch normalization. In brief, the architecture of our network consisted in five 1D-convolutional layers with a kernel size of 2, except for the second layer of varying kernel sizes as stated above (the first layer had 250 neurons, the second 20 neurons, and the remaining 125 neurons each). These convolutional layers were intercalated with batch normalization and followed by an average pooling step. Thereafter, two fully connected layers with 125 neurons intercalated with 50% dropout were included. Finally, we included an output layer using a softmax function to estimate the probability for each model. We used minibatches of size 500 and rectified linear unit activation functions (i.e., ReLUs[4]). Network weights were updated with Adam optimization procedure[5] and the network performance was assessed with a categorical cross-entropy loss function. To avoid overfitting, we used two approaches based on the accuracy for the validation set: model checkpoint that saved only the best model and early stopping, which tolerated a maximum of 150 epochs without any improvement in the validation set accuracy. The trained model was calibrated using temperature scaling[6], with a modified version of the scripts provided by Kull et al.[7] (available at https://github.com/markus93/NN_calibration). The trained network for each species was used to predict the most likely model on 100 randomly sampled datasets of 1,000 SNPs from the empirical data and on a new set of 10,000 independent simulations per scenario (test set), not evaluated by the network during the training. Such predictions were then used to perform an ABC step , using an approach similar to Mondal et al.[8] and also recommended by Sanchez et al.[3]. Cross-validation runs were performed with 10 pseudo-observed simulations per scenario to evaluate the capacity of our ABC implementation to predict the correct simulated scenario from the test set CNN predictions (10,000 simulations per scenario). The CNN predictions for the empirical data were averaged and used as input to perform a rejection step retaining the 5% (threshold selected after a trial run with 5 different thresholds in the *Euphorbia seguieriana* dataset) more similar simulations to approximate the posterior using the rejection algorithm implemented in the R package 'abc'[9]. The same procedure was applied to perform parameter estimation, with the difference that only simulations for the preferred model were used and only 0.1% (threshold also selected after trial runs in the *E. seguieriana* dataset) of them were retained in the posterior. All scripts used to perform our CNN and ABC approaches are available at: https://github.com/manolofperez/CNN_ABCsteppe.

Palynological record

Palynological data have been downloaded from the EPD (European Pollen Database; http://www.europeanpollendatabase.net/data/) and PANGAEA database (https://www.pangaea.de/) and are freely available on these websites. Percentages of arboreal and non-arboreal pollen grains have been calculated for each pollen site (metadata shown in Supplementary Table 4). These results represent changes in forest and open land proportions through time. A synthetic pollen diagram has been done by averaging by time windows the percentages of arboreal and non-arboreal pollen grains falling into a common time interval (Figure 3). Large time windows of 6000 years have been used to reduce potential uncertainties in combining pollen records through space and time, e.g. dating and age-depth modelling, and pollen data are accordingly shown as barplots of 6000 years (Supplementary Figure 3A). Note that pollen analyses of long sedimentary cores provide unique information about long-term trends in vegetation and environmental changes, over millennia to million years. Very few pollen records are available to cover the last interglacial-glacial cycles. Here, we have collected the longest (in time) pollen records covering the last Quaternary climate cycles in Europe, and in particular the last cycle from about 115 ka yrs (Supplementary Figure 3A).

Pollen-based land cover modelling for the Holocene was taken from Marquer et al.[10] (Supplementary Figure 3B). Specifically, these data correspond to pollen counts from all pollen sites present in a radius of ca. 50 km at specific locations spread over Europe. Pollen counts are summed up by time window intervals 0-100, 100-350, 350-700 BP for the three first time windows, and 500 calendar years each from 700 to 11,700 BP. Pollen counts grouped by time windows are then used to run the REVEALS model[11] (Regional Estimates of VEgetation Abundance from Large Sites) to correct biases in inter-taxonomic differences in pollen production, dispersal and deposition mechanisms at regional spatial scales. The model further considers the size of the sedimentary basins for a more realistic estimation of the pollen deposition. All details about this model and its outcomes are described in Marquer et al.[10,12]. The synthetic diagram showing the relative proportions of forest and open land covers correspond to regions A and B (see Marquer et al.[10]) combined, i.e. The Alps + Central Europe.

Supplementary Table 1. Confusion matrices for the five studied species showing the proportion of simulations predicted for each simulated scenario. The main diagonal, with values presented in bold, represents simulations that were correctly predicted. UL - uncalibrated model loss; CL - calibrated model loss.

S. nigromaculatus (UL = 0.5996; CL = 0.5559)

		Predicted Model		
		No expansion	Parallel Expansion	Zonal expansion only
Simulated Model	No expansion	**0.8708**	0.0189	0.1103
	Parallel Expansion	0.0351	**0.9124**	0.0525
	Zonal expansion only	0.0496	0.1120	**0.8384**

O. petraeus (UL = 0.8907; CL = 0.8231)

		Predicted Model		
		No expansion	Parallel Expansion	Zonal expansion only
Simulated Model	No expansion	**0.7856**	0.0048	0.2096
	Parallel Expansion	0.0012	**0.9987**	0.0001
	Zonal expansion only	0.0147	0.0995	**0.8859**

S. capillata (UL = 0.6745; CL = 0.5777)

		Predicted Model		
		No expansion	Parallel Expansion	Zonal expansion only
Simulated Model	No expansion	**1.0000**	0.0000	0.0000
	Parallel Expansion	0.0069	**0.8691**	0.1240
	Zonal expansion only	0.0176	0.1066	**0.8758**

E. segueriana (UL = 0.1262; CL = 0.1158)

		Predicted Model		
		No expansion	Parallel Expansion	Zonal expansion only
Simulated Model	No expansion	**1.0000**	0.0000	0.0000
	Parallel Expansion	0.0008	**0.9973**	0.0019
	Zonal expansion only	0.0000	0.0088	**0.9992**

P. taurica (UL = 0.8906; CL = 0.8241)

		Predicted Model		
		No expansion	Parallel Expansion	Zonal expansion only
Simulated Model	No expansion	**0.8811**	0.0668	0.0521
	Parallel Expansion	0.0055	**0.8679**	0.1267
	Zonal expansion only	0.1031	0.2278	**0.6691**

Supplementary Table 2. Model selection results using empirical data from the five studied species. Posterior probabilities obtained from ABC are shown for each scenario. The scenario with the highest probability for each method is shown in bold.

Scenario	*S. nigromaculatus*	*O. petraeus*	*S. capillata*	*E. segueriana*	*P. taurica*
Parallel Expansion	**1.000**	**1.000**	**0.992**	0.000	0.040
Zonal expansion only	0.000	0.000	0.000	**1.000**	**0.960**
No expansion	0.000	0.000	0.008	0.000	0.000

Supplementary Table 3. Prior values and parameter estimates obtained from the preferred demographic model for each species. T1 – time of the splitting event between the two lineages; T2 – time for the first demographic event, associated with the LGP; T3 – time for the third demographic event, in the end of the LGP; n1a – effective population size of the extrazonal lineage pre-LGP; n1b – effective population size of the zonal lineage pre-LGP; n2a – effective population size of the extrazonal lineage during LGP; n2b – effective population size of the zonal lineage during LGP; n3a – current effective population size of the extrazonal lineage; n3b – current effective population size of the zonal lineage; m1_Z-ExZ – effective number of migrants per generation from zonal to extrazonal lineage pre-LGP; m1_ExZ-Z – effective number of migrants per generation from extrazonal to zonal lineage pre-LGP; m2_Z-ExZ – effective number of migrants per generation from zonal to extrazonal lineage during LGP; m2_ExZ-Z – effective number of migrants per generation from extrazonal to zonal lineage during LGP.

	S. nigromaculatus				*O. petraeus*				*S. capillata*				*E. segueriana*				*P. taurica*			
Parameter	Prior	Median	95%HPD	Error	Prior	Median	95%HPD	Error	Prior	Median	95%HPD	Error	Prior	Median	95%HPD	Error	Prior	Median	95%HPD	Error
T1 (kya)	110-2400	1233.6	495.9-1942.7	1.12	110-2400	1329.4	315.6-2366.7	1.09	110-2400	1608.3	801-2019.1	1.82	110-2400	912.7	318.3-2246.4	1.25	110-2400	977.3	294.3-1783.8	2.03
T2 (kya)	12-110	51.0	27.8-99.4	0.92	12-110	54.2	19.8-89.2	0.64	12-110	69.8	23.8-102.5	1.20	12-110	81.9	15.6-100.8	2.55	12-110	68.5	18.7-107.6	0.98
T3 (kya)	0-12	3.5	0.4-10.1	1.30	0-12	3.2	0.4-8.5	1.08	0-12	6.7	0.6-10.5	1.72	0-12	6.1	3.2-10.8	0.95	0-12	5.3	4.1-9.7	0.72
n1a (k)	10-100	36.6	24.9-49.1	1.22	10-200	90.6	43.3-160.5	1.08	5-100	25.9	12-71.6	1.06	-	-	-	-	-	-	-	-
n1b (k)	10-100	36.7	29-57.9	1.95	10-200	124.1	45.7-164.7	1.57	5-100	30.9	10.3-61.5	0.97	20-200	106.2	48.2-130.9	1.08	50-600	227.9	90.5-427	0.88
n2a (k)	100-10000	4654.3	891.6-6788.8	1.35	100-20000	9540.8	2117.6-17728.8	1.01	50-10000	1379.0	443.4-6435.3	1.21	-	-	-	-	-	-	-	-
n2b (k)	100-10000	2804.1	795.9-6359.5	1.04	100-20000	8306.6	2565.2-13738.6	1.08	50-10000	1448.8	388.2-7804.3	1.27	200-20000	8280.7	1649.9-17312.3	1.20	500-60000	9161.4	2337.2-22004.7	1.11
n3a (k)	20-100	56.7	33.2-79.6	1.53	20-200	156.3	54.2-181.5	1.49	10-100	36.0	14.5-85.3	0.91	40-200	126.8	72.4-192.2	1.15	100-600	333.3	112.5-572.3	0.94
n3b (k)	4-500	161.3	22.9-229.8	0.77	4-1000	516.7	117.6-823.9	1.43	2-500	96.7	10.3-295.5	1.51	8-1000	274.1	53.6-566	0.83	20-3000	586.7	218-2176.1	0.87
m1_Z-ExZ	0-5	2.00	0.4-4	1.75	0-5	2.12	0.1-4.1	1.26	0-5	3.37	1.1-4.6	1.26	0-5	2.30	0.2-4.6	0.95	0-5	2.85	1.4-4.6	1.66
m1_ExZ-Z	0-5	1.96	0.4-4.5	0.69	0-5	2.85	0.4-4.7	0.72	0-5	3.01	1.2-5	1.22	0-5	2.63	0.2-4.5	0.96	0-5	2.67	0.6-4.9	1.06
m2_Z-ExZ	0-5	2.09	0.1-4.9	1.23	0-5	4.11	0.2-4.9	1.11	0-5	1.85	0.3-4	1.86	0-5	3.44	1.3-4.5	0.78	0-5	1.89	0.7-4.7	0.78
m2_ExZ-Z	0-5	2.16	0.1-3.8	0.80	0-5	3.61	0.9-4.8	1.26	0-5	1.49	0.4-4.8	1.37	0-5	2.16	0.3-4.6	0.87	0-5	2.85	0.4-4.3	1.39

Supplementary Table 4. Metadata for EPD: European Pollen Database.

**Site Name**	**Data source**	**Lat.**	**Long.**	**Elevation (m)**	**Chronology (ky BP)**	**Publications**
Ioannina	EPD	39°40′N	20°51′E	470	ca. 522 to 6	Tzedakis[13], Tzedakis et al.[14]
Lake Ohrid	PANGAEA	40 °54 to 41° 10N	20°38 to 20°48E	693	ca. 501 to 1	Sadori et al.[15]
Lago Grande di Monticchio	PANGAEA	40°56′N	15°36′E	656	ca. 101 to present	Allen et al.[16,17]
La Grande Pile	EPD<br>PANGAEA	47°44′N	6°30′E	330	ca. 135 to 11	de Beaulieu and Reille[18]
Lac du Bouchet	EPD	44°50′N	3°47′E	1200	ca. 123 to 4	Reille and de Beaulieu[19]

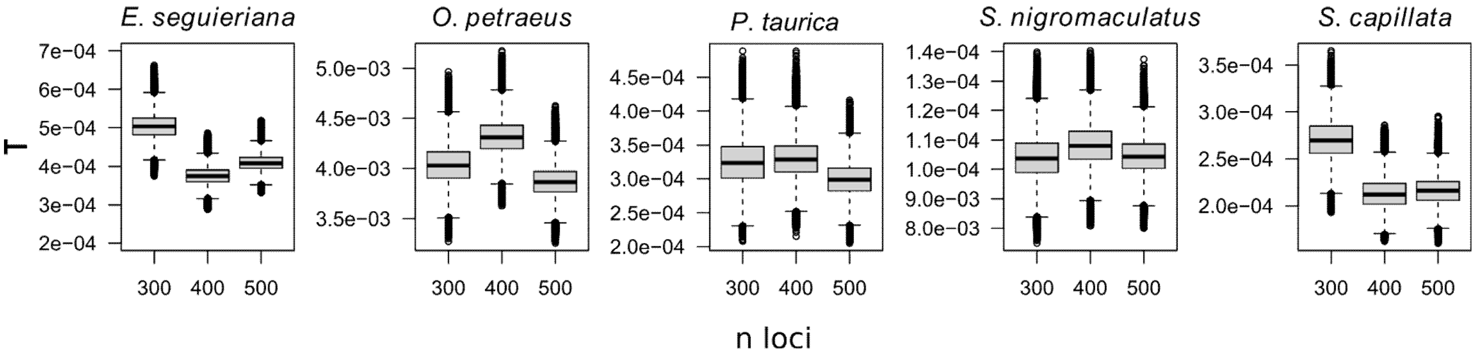

Supplementary Figure 1. Species specific τ estimates for different subsets of SNPs (300,400,500) obtained via multi-species coalescent based inference in bpp[20]. Boxplots are based on the posterior distribution of τ estimates from at least 1,000,000 generations (i.e. number of generations after 100,000 generations were discarded as burnin); boxes show Q1 to Q3 interquartile range (IQR; 25th to 75th percentile), the center line depicts the median, whiskers show minima and maxima defined as Q1-1.5*IQR and Q3+1.5*IQR respectively. Source data are provided as a Source Data file.

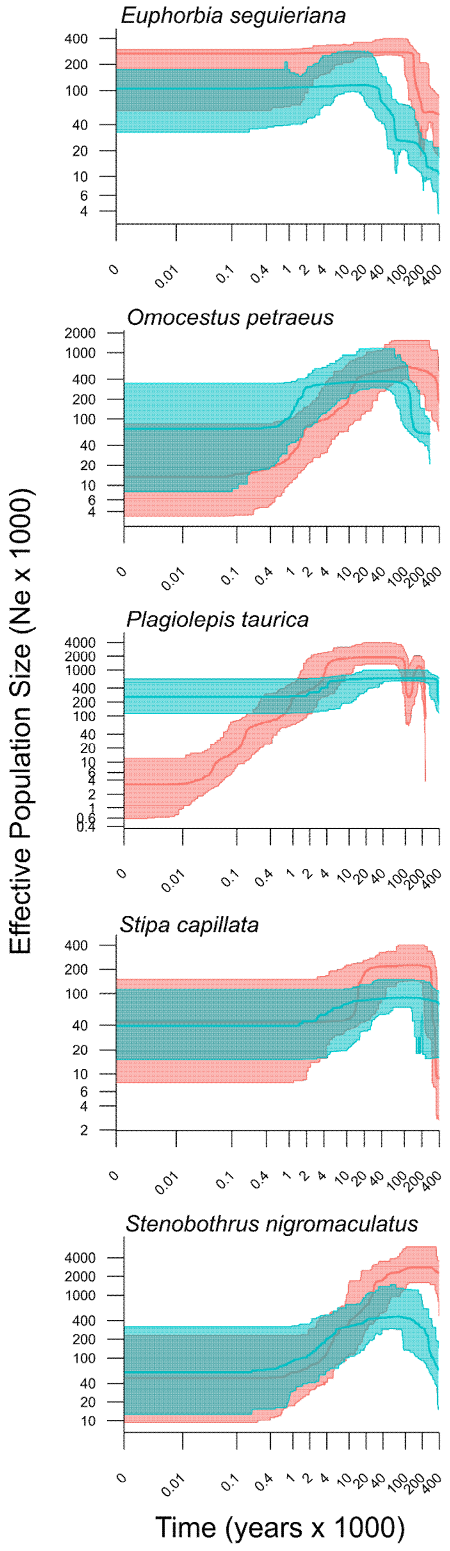

Supplementary Figure 2. Stairway plots representing the change in effective population size (Ne) over time (kya) for each species. Stairway plots from zonal lineages are shown in blue, those from extrazonal lineages in red. The thick lines represent the median Ne, and confidence intervals (2.5, 97.5 percentiles) are represented by the transparent polygon in the respective color. Ne and kya values were log-transformed for better visualization of recent changes, and x- and y-axes in the graphs were

correspondingly adapted. Source data are provided as a Source Data file.

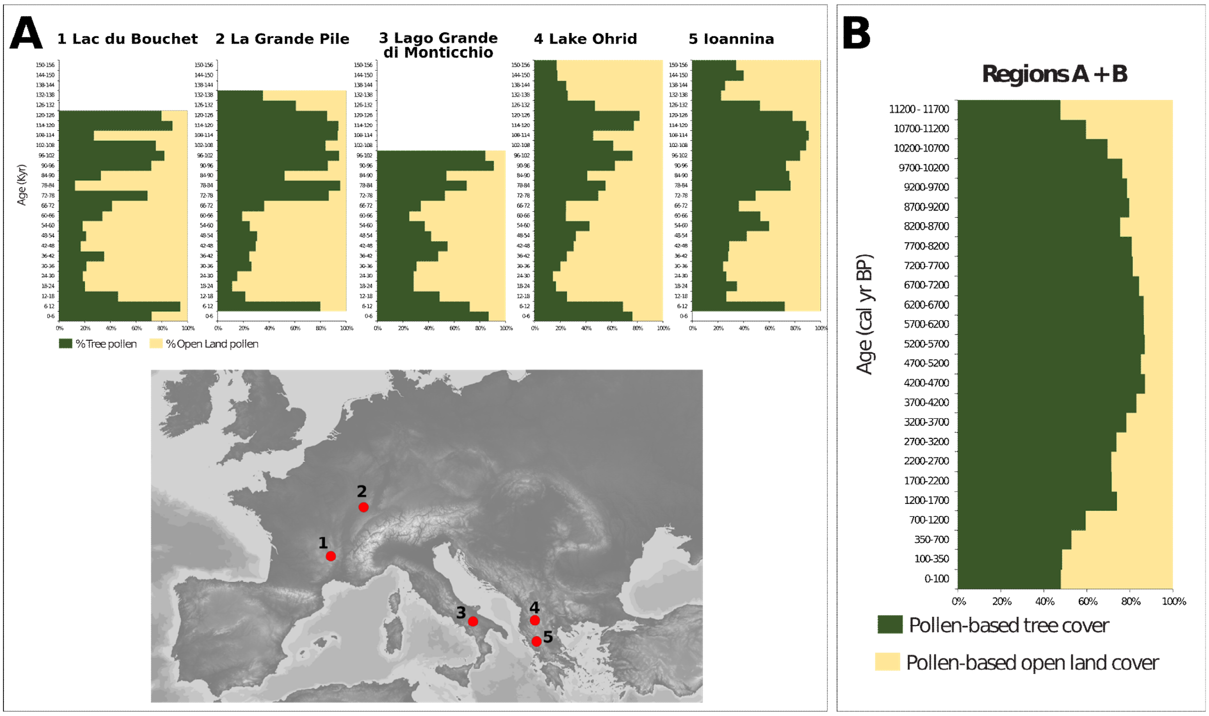

Supplementary Figure 3: Forest (green) and open land (yellow) pollen data for the last glacial-interglacial cycle with a zoom in for the Holocene. Details in Supplementary Methods. **A.** Pollen percentages data from five major pollen records covering the entire last glacial-interglacial cycle, and their geographic origin. All chronologies are expressed in age BP and are based on the original publications (Supplementary Table 4). **B.** Pollen-based land cover modelling for the Holocene as in Marquer et al.[10]. Source data are provided as a Source Data file.

References

1. Hudson, R. R. Generating samples under a Wright–Fisher neutral model of genetic variation. *Bioinformatics* **18**, 337–338 (2002).
2. Oliveira, E. A. *et al.* Historical demography and climate driven distributional changes in a widespread Neotropical freshwater species with high economic importance. *Ecography* **43**, 1291–1304 (2020).
3. Sanchez, T., Cury, J., Charpiat, G. & Jay, F. Deep learning for population size history inference: Design, comparison and combination with approximate Bayesian computation. *Mol. Ecol. Resour.* **00**, 1–16 (2020).
4. Nair, V. & Hinton, G. E. Rectified Linear Units Improve Restricted Boltzmann Machines. *Proc. 27th Int. Conf. Mach. Learn. ICML-10* 807–814 (2010).
5. Kingma, D. P. & Ba, J. Adam: A Method for Stochastic Optimization. *ArXiv 14126980* (2014).
6. Guo, C., Pleiss, G., Sun, Y. & Weinberger, K. Q. On Calibration of Modern Neural Networks. *ArXiv170604599 Cs* (2017).
7. Kull, M. *et al.* Beyond temperature scaling: Obtaining well-calibrated multiclass probabilities with Dirichlet calibration. *ArXiv191012656 Cs Stat* (2019).
8. Mondal, M., Bertranpetit, J. & Lao, O. Approximate Bayesian computation with deep learning supports a third archaic introgression in Asia and Oceania. *Nat. Commun.* **10**, 246 (2019).
9. Csilléry, K., François, O. & Blum, M. G. B. abc: an R package for approximate Bayesian computation (ABC). *Methods Ecol. Evol.* **3**, 475–479 (2012).
10. Marquer, L. *et al.* Quantifying the effects of land use and climate on Holocene vegetation in Europe. *Quat. Sci. Rev.* **171**, 20–37 (2017).
11. Sugita, S. Theory of quantitative reconstruction of vegetation I: pollen from large sites REVEALS regional vegetation composition. *The Holocene* **17**, 229–241 (2007).
12. Marquer, L. *et al.* Holocene changes in vegetation composition in northern Europe: why quantitative pollen-based vegetation reconstructions matter. *Quat. Sci. Rev.* **90**, 199–216 (2014).
13. Tzedakis, P. C. Long-term tree populations in northwest Greece through multiple Quaternary climatic cycles. *Nature* **364**, 437–440 (1993).
14. Tzedakis, P. C., Bennett, K. D., Magri, D. & Magri, D. Climate and the pollen record. *Nature* **370**, 513–513 (1994).

15. Sadori, L. *et al.* Pollen-based paleoenvironmental and paleoclimatic change at Lake Ohrid (south-eastern Europe) during the past 500 ka. *Biogeosciences* **13**, 1423–1437 (2016).
16. Allen, J. R. M., Watts, W. A. & Huntley, B. Weichselian palynostratigraphy, palaeovegetation and palaeoenvironment; the record from Lago Grande di Monticchio, southern Italy. *Quat. Int.* **73–74**, 91–110 (2000).
17. Allen, J. R. M. *et al.* Rapid environmental changes in southern Europe during the last glacial period. *Nature* **400**, 740–743 (1999).
18. de Beaulieu, J.-L. & Reille, M. Long Pleistocene pollen sequences from the Velay Plateau (Massif Central, France). *Veg. Hist. Archaeobotany* **1**, 233–242 (1992).
19. Reille, M. & de Beaulieu, J. L. Pollen analysis of a long upper Pleistocene continental sequence in a Velay maar (Massif Central, France). *Palaeogeogr. Palaeoclimatol. Palaeoecol.* **80**, 35–48 (1990).
20. Flouri, T., Jiao, X., Rannala, B. & Yang, Z. Species tree inference with BPP using genomic sequences and the multispecies coalescent. *Mol. Biol. Evol.* **35**, 2585–2593 (2018).
