# Supplementary material for: Congruent evolutionary responses of European steppe biota to late Quaternary climate change
Source: Nat Commun. 2022 Apr 8;13:1921. doi: 10.1038/s41467-022-29267-8 (PMC8993823; doi:10.1038/s41467-022-29267-8)
Supplement: Supplementary file 3 — Description of Additional Supplementary Information [file 41467_2022_29267_MOESM3_ESM.pdf]

Description of Additional Supplementary Files

Title: Supplementary Data 1

Description: The Supplementary Data file is a table that contains Individual Ids, Populations IDs, sampling localities, NCBI SRA accession codes etc.
