# Supplementary material for: Congruent evolutionary responses of European steppe biota to late Quaternary climate change
Source: Nat Commun. 2022 Apr 8;13:1921. doi: 10.1038/s41467-022-29267-8 (PMC8993823; doi:10.1038/s41467-022-29267-8)
Supplement: Supplementary file 4 — Reporting Summary [file 41467_2022_29267_MOESM4_ESM.pdf]

nature portfolio

Corresponding author(s): Philipp Kirschner

Last updated by author(s): Feb 3, 2022

Reporting Summary

Nature Portfolio wishes to improve the reproducibility of the work that we publish. This form provides structure for consistency and transparency in reporting. For further information on Nature Portfolio policies, see our Editorial Policies and the Editorial Policy Checklist.

Statistics

For all statistical analyses, confirm that the following items are present in the figure legend, table legend, main text, or Methods section.

n/a	Confirmed	
☐	☒	The exact sample size (n) for each experimental group/condition, given as a discrete number and unit of measurement
☒	☐	A statement on whether measurements were taken from distinct samples or whether the same sample was measured repeatedly
☒	☐	The statistical test(s) used AND whether they are one- or two-sided<br>*Only common tests should be described solely by name; describe more complex techniques in the Methods section.*
☒	☐	A description of all covariates tested
☒	☐	A description of any assumptions or corrections, such as tests of normality and adjustment for multiple comparisons
☐	☒	A full description of the statistical parameters including central tendency (e.g. means) or other basic estimates (e.g. regression coefficient) AND variation (e.g. standard deviation) or associated estimates of uncertainty (e.g. confidence intervals)
☒	☐	For null hypothesis testing, the test statistic (e.g. F, t, r) with confidence intervals, effect sizes, degrees of freedom and P value noted<br>*Give P values as exact values whenever suitable.*
☐	☒	For Bayesian analysis, information on the choice of priors and Markov chain Monte Carlo settings
☒	☐	For hierarchical and complex designs, identification of the appropriate level for tests and full reporting of outcomes
☒	☐	Estimates of effect sizes (e.g. Cohen's d, Pearson's r), indicating how they were calculated

Our web collection on statistics for biologists contains articles on many of the points above.

Software and code

Policy information about availability of computer code

Data collection	No software was used for data collection.
Data analysis	Software: R version 3.6.3; Stacks 2.3d; STRUCTURE v. 2.3.4.; BPP v. 4.2.9; fasta2genotype.py (no information on version available; downloaded from github.com/paulmaier/fasta2genotype on 29th July 2020); Tracer v. 1.6.0; bppr R package v. 0.6.1; easySFS.py (no information on version available; downloaded from github.com/isaacovercast/easySFS on 11th May 2020); Stairway Plot v. 2.1; ms; phylospatial; QGIS v. 3.10; Spectral Profile Tool v. 2.0.3 in QGIS v. 3.10; abc R package; vcftools v. 0.1.16;<br><br>Custom code as used for the convolutional neural network approach is available as mentioned in the coda availability statement (https://github.com/manolofperez/CNN_ABCsteppe.git)

For manuscripts utilizing custom algorithms or software that are central to the research but not yet described in published literature, software must be made available to editors and reviewers. We strongly encourage code deposition in a community repository (e.g. GitHub). See the Nature Portfolio guidelines for submitting code & software for further information.

Data

Policy information about availability of data

All manuscripts must include a data availability statement. This statement should provide the following information, where applicable:

- Accession codes, unique identifiers, or web links for publicly available datasets
- A description of any restrictions on data availability
- For clinical datasets or third party data, please ensure that the statement adheres to our policy

Demultiplexed RADseq sequencing data are available from the NCBI GenBank Short Read Archive. All accession numbers are given in Supplementary Data 1. The sources of all pollen data are cited in Supplementary Table 4, and the used data is provided in the Source Data for Supplementary Figure 3.

Field-specific reporting

Please select the one below that is the best fit for your research. If you are not sure, read the appropriate sections before making your selection.

☐ Life sciences ☐ Behavioural & social sciences ☒ Ecological, evolutionary & environmental sciences

For a reference copy of the document with all sections, see nature.com/documents/nr-reporting-summary-flat.pdf

Ecological, evolutionary & environmental sciences study design

All studies must disclose on these points even when the disclosure is negative.

Study description	We tested which of several demographic scenarios captures the late Quaternary range dynamics of five European steppe species (2 vascular plants, 3 insects) best. This was done based on genomic RADseq data generated in a previous study, and by using an approach combining convolutional neural networks and approximate Bayesian computation. We used exploratory demographic analyses based on the site frequency spectrum, and multi-species coalescent models to infer priors for the employed model. Modeling results were complemented niche models for the last glacial maximum, and publicly available climate and paleoecological data available for the modeled time period.
Research sample	The research sample consists of RADseq data, ecological niche models and occurrence data from five typical species of dry-continental Eurasian steppes (Euphorbia seguieriana, Stipa capillata, Omocestus petraeus, Plagiolepis taurica, Stenobothrus nigromaculatus). As specified in the Methods section, the data analyzed in this study was generated by Kirschner & Zaveska et al. (Nat Commun 11, 1968; 2020), and has been made available by these authors via the NCBI short read archive. Populations were defined as a set of individuals collected from the same locality (given as locality ID in Supplementary Data 1).
Sampling strategy	We used raw data that was generated in Kirschner & Zaveska et al. (Nat Commun 11, 1968; 2020). These data capture a large number of samples from large parts of the species' European distributions, and are considered to be representative for the European steppes. No additional data that would complement these data were available when writing the manuscript.<br>The rationale behind the selection of pollen data is elaborated in the Supplementary Information. In short, the longest (in time) pollen records covering the last quaternary climate cycles in Europe were selected.
Data collection	RADseq was downloaded from the NCBI short read archive (accessions given in Supplementary Data 1). Species occurrence data and niche models are available from source data given in Kirschner & Zaveska et al (Nat Commun 11, 1968; 2020). Collectors are listed in Supplementary Data 1. Pollen data has been downloaded from the European Pollen Database and the PANGEA database (specified in Supplementary Information & Supplementary Table 4; European Pollen Database; http://www.europeanpollendatabase.net/data/, PANGAEA database (https://www.pangaea.de/). Pollen based land cover data for the Holocene was taken from Marquer et al. (Quat. Sci. Rev. 171; 2017). Temperature time series data as shown in Figure 3 was taken from Hansen et al. (Phil. Trans. R. Soc. A. 371; 2013) and is publicly available.
Timing and spatial scale	All samples were collected between 2014 and 2016 with earlier sampling for trial analyses (21.6.2001 - 23.10.2015). The sampling period has no influence on the analytical approaches applied here, and was thus considered irrelevant. The area of sampling reflects large parts of the Eurasian steppe biome.
Data exclusions	No data were excluded from the analyses
Reproducibility	All findings are reproducible given the presented methodology and data. Attempts to reproduce the presented results were successful.
Randomization	As our study was based on randomly collected field samples, no randomization was necessary.
Blinding	Blinding was not necessary for this study

Did the study involve field work? ☐ Yes ☒ No

Reporting for specific materials, systems and methods

We require information from authors about some types of materials, experimental systems and methods used in many studies. Here, indicate whether each material, system or method listed is relevant to your study. If you are not sure if a list item applies to your research, read the appropriate section before selecting a response.

Materials & experimental systems

n/a	Involved in the study
☒	☐ Antibodies
☒	☐ Eukaryotic cell lines
☒	☐ Palaeontology and archaeology
☒	☐ Animals and other organisms
☒	☐ Human research participants
☒	☐ Clinical data
☒	☐ Dual use research of concern

Methods

n/a	Involved in the study
☒	☐ ChIP-seq
☒	☐ Flow cytometry
☒	☐ MRI-based neuroimaging
